# Supplementary material for: The Combined Effects of an External Field and Novel Functional Groups on the Structural and Electronic Properties of TMDs/Ti3C2 Heterostructures: A First-Principles Study
Source: Nanomaterials (Basel). 2023 Mar 29;13(7):1218. doi: 10.3390/nano13071218 (PMC10097373; doi:10.3390/nano13071218)
Supplement: Supplementary file 1 [file nanomaterials-13-01218-s001.zip › nanomaterials-2256366-supplementary.pdf]

Supplementary Materials

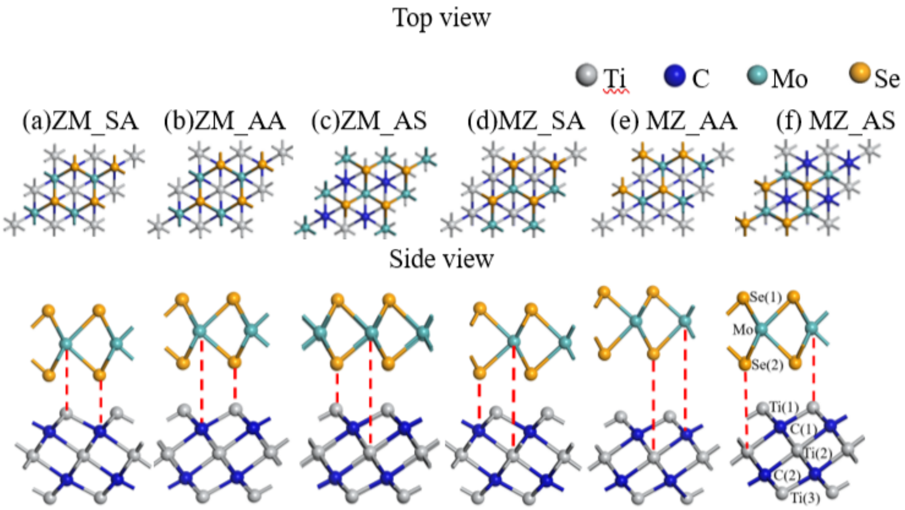

Figure S1. A schematic diagram of top and side views of $MoSe_2/Ti_3C_2$ heterostructures for different stackings. (**a**) the ZM_SA Configuration: Se and Mo atoms of $MoSe_2$ are on top of Ti and C atoms of Ti_3C_2, respectively; (**b**) the ZM_AA Configuration: Se and Mo atoms of $MoSe_2$ are on top of C and Ti atoms of Ti_3C_2, respectively; (**c**) the ZM_AS Configuration: Se and Mo atoms of $MoSe_2$ are on top of Ti atoms and hollow sites of Ti_3C_2, respectively; (**d**) the MZ_SA Configuration: Se and Mo atoms of $MoSe_2$ are on top of C atoms and hollow sites of Ti_3C_2, respectively; (**e**) the MZ_AA Configuration: Mo and Se atoms of $MoSe_2$ are on top of C atoms and hollow sites of Ti_3C_2, respectively; (**f**) the MZ_AS Configuration: Mo and Se atoms of $MoSe_2$ are on top of Ti atoms and hollow sites of Ti_3C_2, respectively.

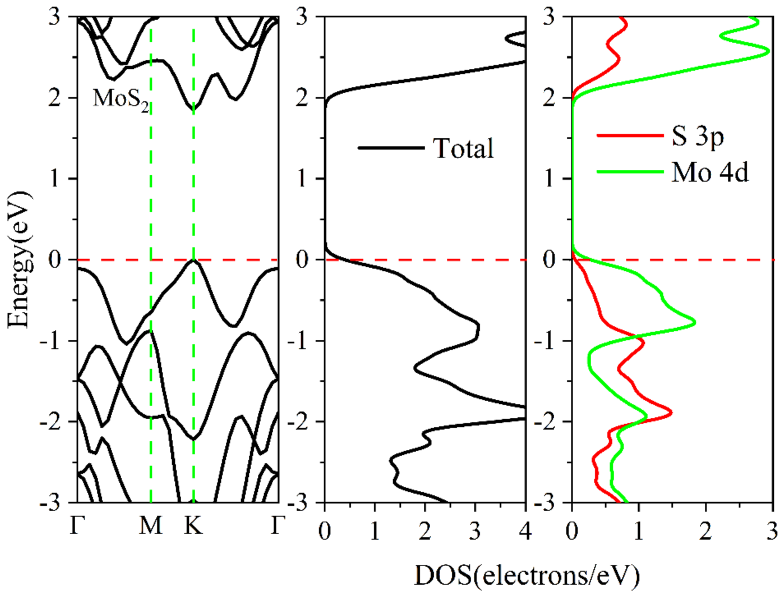

Figure S2. Band structures and densities of states of the MoS_2. The Fermi level is set to 0 eV. The vertical dashed line gives the location of the Fermi level.

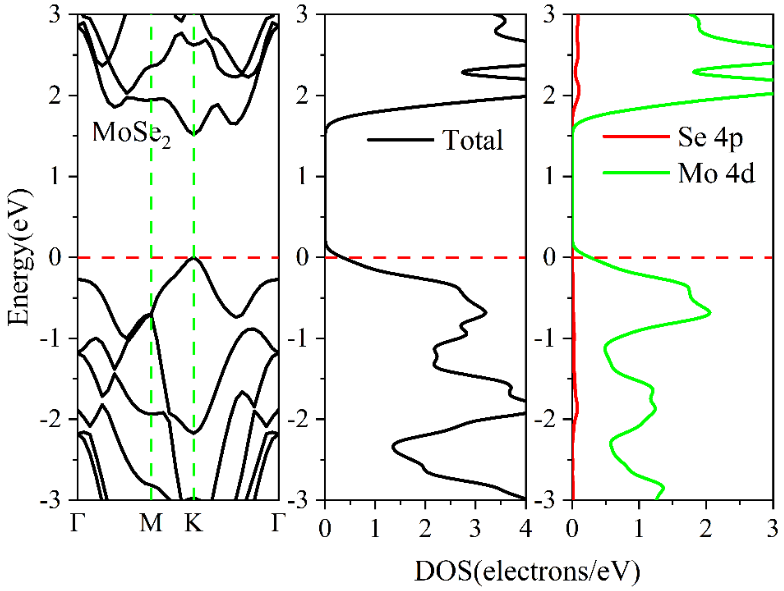

Figure S3. Band structures and densities of states of the $MoSe_2$. The Fermi level is set to 0 eV. The vertical dashed line gives the location of the Fermi level.

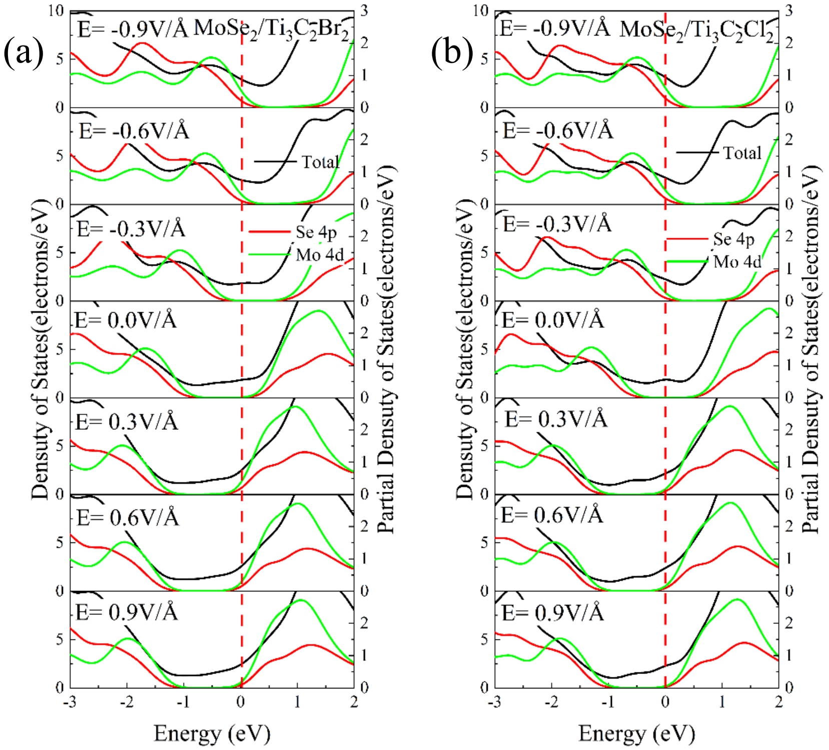

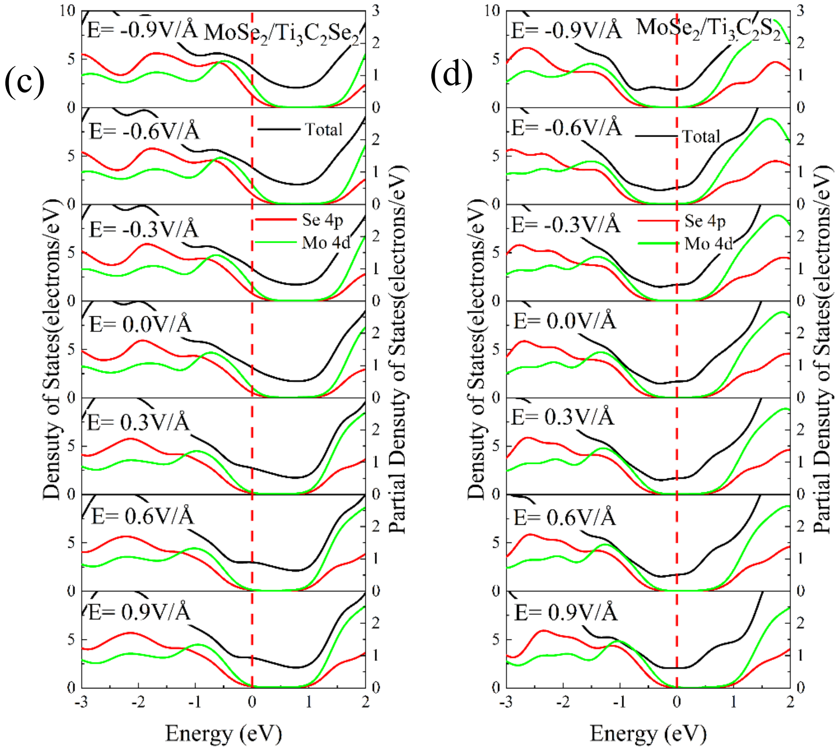

Figure S4. The DOS of $MoSe_2/Ti_3C_2X_2$ and PDOS of $MoSe_2$ in $MoSe_2/Ti_3C_2X_2$ heterostructures under different electric fields. (**a**) X-Br, (**b**) X-Cl, (**c**) X-Se and (**d**) X-S.

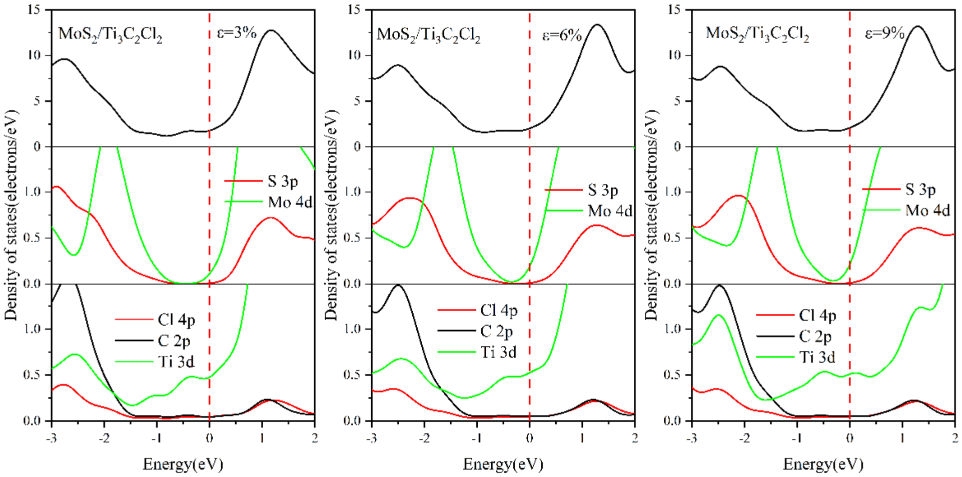

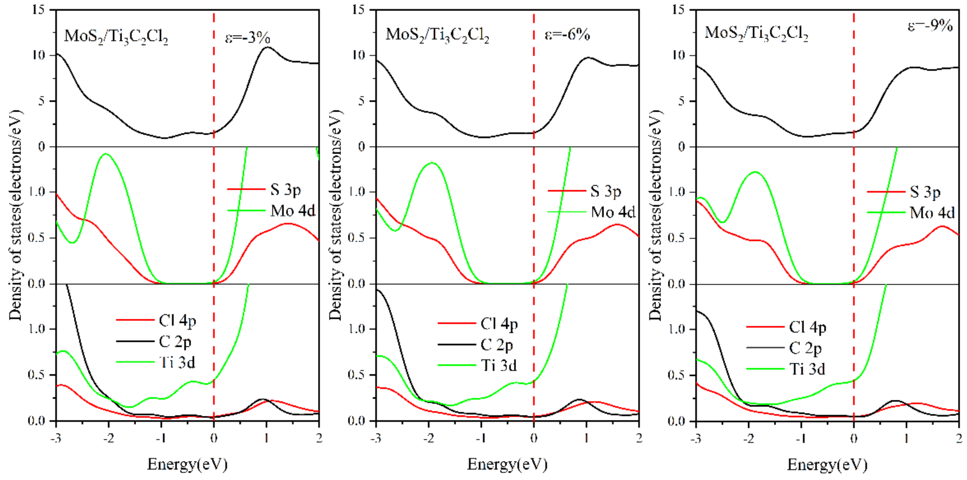

Figure S5. Density of states of the $MoS_2/Ti_3C_2Cl_2$ heterostructure with different biaxial strains. The Fermi level is set to 0 eV.

Figure S5 shows the DOS of $MoS_2/Ti_3C_2Cl_2$ heterostructure, under tensile and compressive strain, the Fermi energy level is always located at the conduction band minimum (CBM) of MoS_2. Under compressive strain, the monolayer MoS_2 retains its band gap. While when the tensile strain reaches to 6%, the band gap of monolayer MoS_2 disappears. It indicates that the tensile strain can significantly improve the interaction strength between the monolayer MoS_2 and $Ti_3C_2Cl_2$.

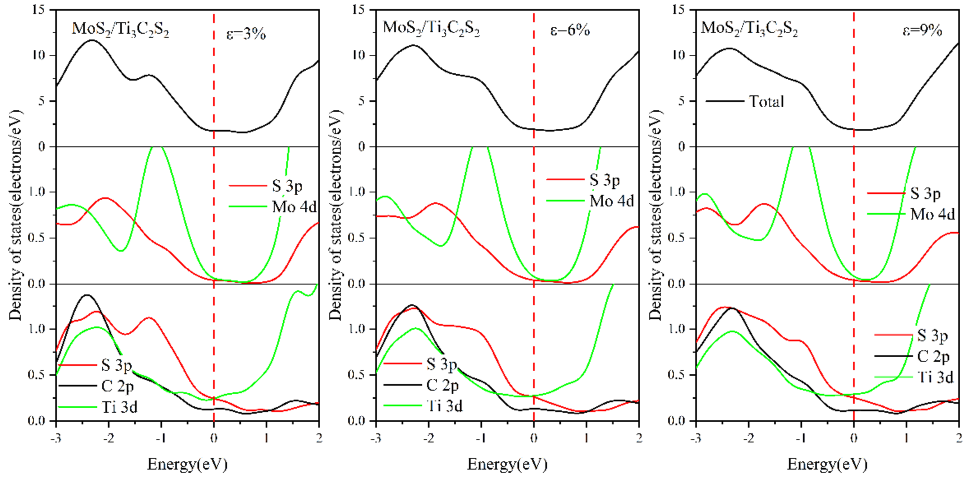

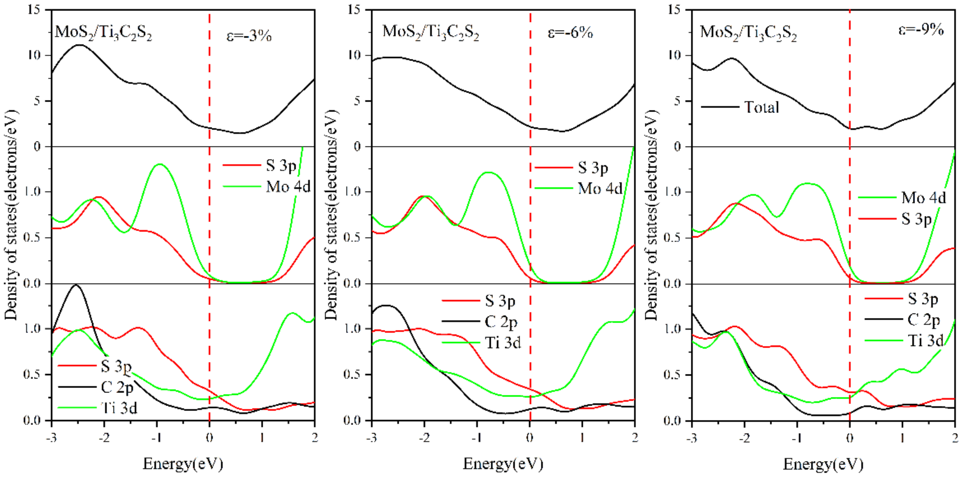

Figure S6. Density of states of the $MoS_2/Ti_3C_2S_2$ heterostructure with different biaxial strains. The Fermi level is set to 0 eV.

According to the DOS of $MoS_2/Ti_3C_2S_2$ heterostructure (see Figure S6), with the increase of compressive strain, the monolayer MoS_2 can still preserve its band gap, while its band gap disappears when the tensile strain is applied, which is very similar to the $MoS_2/Ti_3C_2Cl_2$ heterostructure.

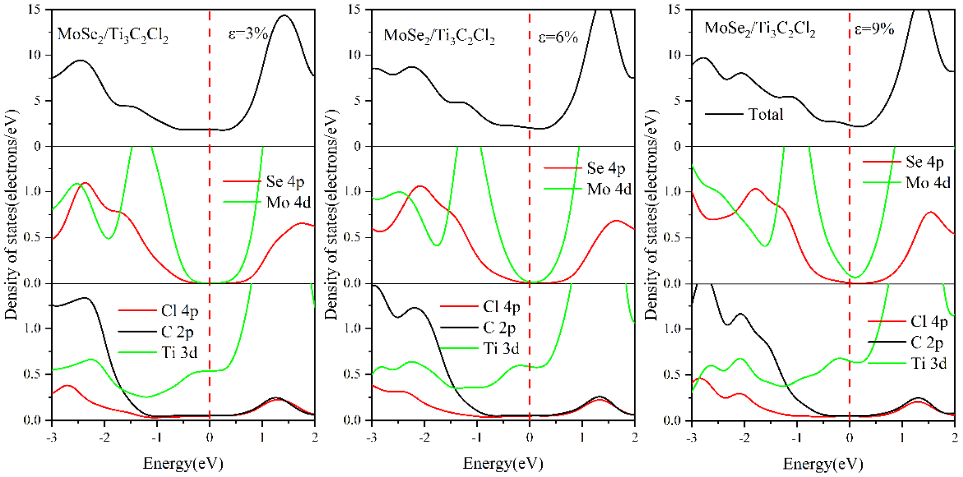

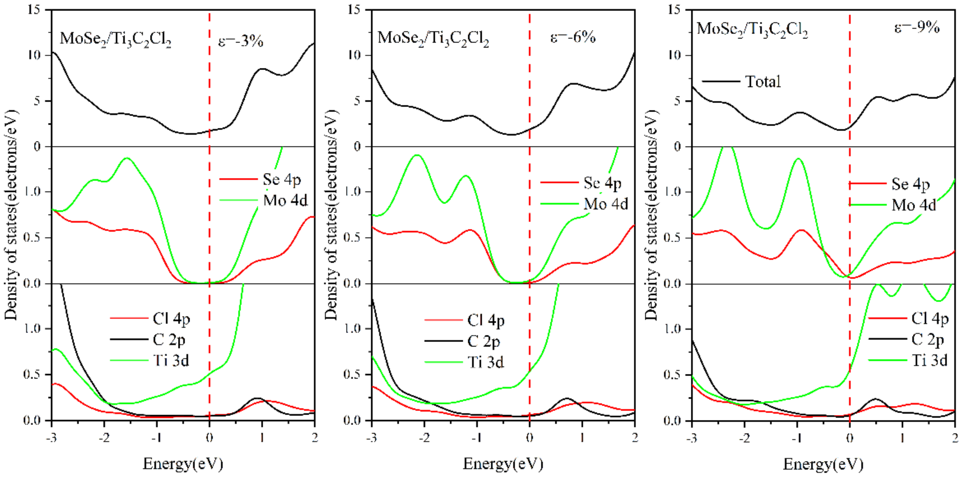

Figure S7. Density of states of the $MoSe_2/Ti_3C_2Cl_2$ heterostructure with different biaxial strains. The Fermi level is set to 0 eV.

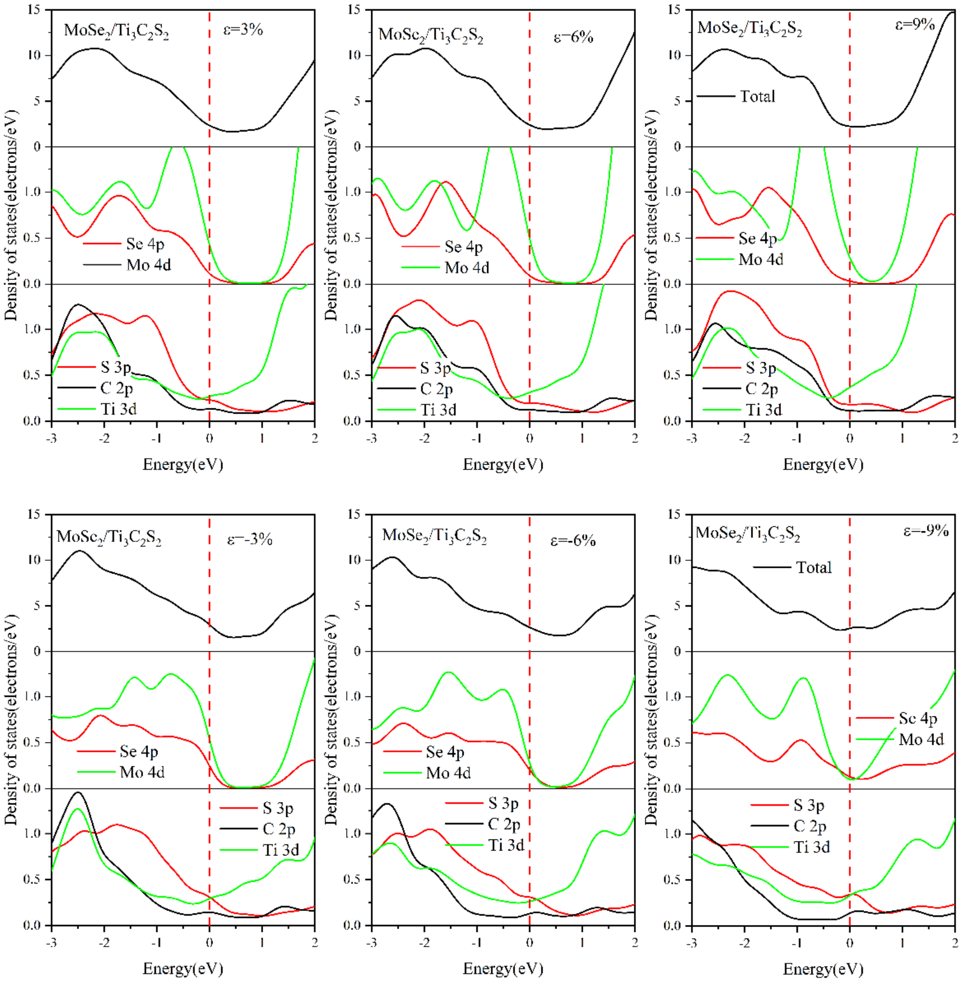

Figure S8. Density of states of the $MoSe_2/Ti_3C_2S_2$ heterostructure with different biaxial strains. The Fermi level is set to 0 eV.

Table S1. Optimized structural parameters for the TMDs/$Ti_3C_2X_2$ (X = S, Se, Br, Cl) heterostructure. S1 and Se1 are surface functional groups.

**Formula**	**Atomic positions**
MoS_2/Ti_3C_2	Ti1 (0,0,0.573) Ti2 (2/3,1/3,0.496) C (1/3,2/3,0.529)<br>Mo (0.338,0.673,0.361) S (0.671,0.34,0.414)
$MoS_2/Ti_3C_2Br_2$	Ti1 (0,0,0.405) Ti2 (2/3,1/3,0.328) C (1/3,2/3,0.363)<br>Br (0.5,0.5,0.541) Mo (1/3,2/3,0.695) S (2/3,1/3,0.644)
$MoS_2/Ti_3C_2Cl_2$	Ti1 (0,0,0.399) Ti2 (2/3,1/3,0.323) C (1/3,2/3,0.363)<br>Cl (1.0,0.0,0.531) Mo (1/3,2/3,0.684) S (2/3,1/3,0.632)
$MoS_2/Ti_3C_2S_2$	Ti1 (0,0,0.399) Ti2 (2/3,1/3,0.318) C (1/3,2/3,0.358)<br>S1 (1.0,0.0,0.533) Mo (0.314,0.634,0.678) S (0.648,0.301,0.627)
$MoS_2/Ti_3C_2Se_2$	Ti1 (0,0,0.61) Ti2 (2/3,1/3,0.531) C (1/3,2/3,0.569)<br>Se1 (1.0,0.0,0.747) Mo (0.336,0.667,0.33) S (0.671,0.334,0.381)
$MoSe_2/Ti_3C_2$	Ti1 (0,0,0.675) Ti2 (2/3,1/3,0.761) C (1/3,2/3,0.725)<br>Mo (1/3,2/3,0.489) Se (2/3,0.1/3,0.424)
$MoSe_2/Ti_3C_2Br_2$	Ti1 (0,0,0.619) Ti2 (2/3,1/3,0.328) C (1/3,2/3,0.659)<br>Br (0.5,0.5,0.488) Mo (1/3,2/3,0.695) Se (2/3,1/3,0.338)
$MoSe_2/Ti_3C_2S_2$	Ti1 (0,0,0.4) Ti2 (2/3,1/3,0.319) C (1/3,2/3,0.359)<br>S1 (1.0,0.0,0.532) Mo (0.314,0.634,0.69) Se (0.648,0.301,0.634)
$MoSe_2/Ti_3C_2Cl_2$	Ti1 (0,0,0.63) Ti2 (2/3,1/3,0.554) C (1/3,2/3,0.589)<br>Cl (0.0,1.0,0.498) Mo (2/3,1/3,0.338) Se (2/3,1/3,0.393)
$MoSe_2/Ti_3C_2Se_2$	Ti1 (0,0,0.631) Ti2 (2/3,1/3,0.552) C (1/3,2/3,0.589)<br>Se1 (1.0,0.0,0.769) Mo (1/3,2/3,0.32) Se (2/3,1/3,0.373)

Table S2. Mulliken charge (electron), bond length (Å) and bond populations of MoS_2/Ti_3C_2.

**Species**	**Charge (*e*)**	**Bond Lengths (bond populations)**
C(1)	−0.75	C–Ti 2.05894 (0.77) 2.06713 (0.90) 2.22905 (0.65) 2.23631(0.25)
C(2)	−0.75	S–Ti 2.46514 (0.48)
S(1)	−0.02	S-Mo 2.39760 (0.91) 2.39760 (0.05) 2.41895 (0.83)
S(2)	−0.03	
Ti(1)	0.42	
Ti(2)	0.38	
Ti(3)	0.75	
Mo	−0.01	

Table S3. Mulliken charge (electron), bond length (Å) and bond populations of $MoSe_2/Ti_3C_2$.

**Species**	**Charge (*e*)**	**Bond Lengths (bond populations)**
C(1)	−0.75	C-Ti 2.06351 (0.80) 2.11855 (0.88) 2.19880 (0.20) 2.26001(0.66)
C(2)	−0.71	Se-Ti 2.69638 (−0.83)
Se(1)	0.14	Se-Mo 2.51199 (1.32) 2.51224 (−0.00) 2.56317 (−0.19)
Se(2)	0.23	
Ti(1)	0.73	
Ti(2)	0.34	
Ti(3)	0.38	
Mo	−0.35	
